# Examining psychometric properties of the Iranian version of exclusive breastfeeding social support scale (EBFSS)

**DOI:** 10.1186/s40359-023-01262-8

**Published:** 2023-08-16

**Authors:** Sepideh Mashayekh-Amiri, Mina Hosseinzadeh, Mohammad Asghari Jafarabadi, Sepideh Soltani, Mojgan Mirghafourvand

**Affiliations:** 1 Students Research Committee, Midwifery Department, Faculty of Nursing and Midwifery, Tabriz University of Medical sciences, Tabriz, Iran; 2 Department of Community Health Nursing, Nursing and Midwifery Faculty, Tabriz University of Medical Sciences, Tabriz, Iran; 3 Cabrini Research, Cabrini Health, Melbourne, VIC 3144 Australia; 4 School of Public Health and Preventative Medicine, Faculty of Medicine, Nursing and Health Sciences, Monash University, Melbourne, VIC 3004 Australia; 5 Road Traffic Injury Research Center, Tabriz University of Medical Sciences, Tabriz, Iran; 6 Students Research Committee, Department of Community Health Nursing, Faculty of Nursing and Midwifery, Tabriz University of Medical sciences, Tabriz, Iran; 7 Social Determinants of Health Research Center, Department of Midwifery, Faculty of Nursing and Midwifery, Tabriz University of Medical Sciences, Tabriz, Iran; 8 Menopause Andropause Research Center, Ahvaz Jundishapur University of Medical Sciences, Ahvaz, Iran

**Keywords:** Validation, Psychometric, Instrument, Exclusive breastfeeding, Social support, EBFSS, Iran

## Abstract

**Background:**

The exclusive breastfeeding (EBF) is undeniably proven significant in mothers’ health and infants’ growth and survival. Its persistence has many familial, social, and economical benefits. Social support is known to be an effective factor in EBF’s success and sustainability. However, Exclusive breastfeeding social support (EBFSS) scale validity and reliability is not evaluated in Iran. This study aimed to determine the psychometric properties of EBFSS during postpartum period in Tabriz city, Iran.

**Methods:**

It is a cross-sectional study with descriptive survey method performed between March 2021 and August 2022. Psychometric properties were determined for the Persian version of EBFSS in six stages: translation process, evaluating content validity, face validity, construct validity, discriminant validity, and reliability. A group of experts (n = 10), followed by a group of women with EBF (n = 10), evaluated the instrument’s items based on content and face validities, respectively. A cross-sectional study using the multi-stage cluster random sampling method on 348 women with EBF in the first four months after delivery was conducted to determine the construct validity. The internal consistency and repeatability (test-retest on 30 women, 2 weeks apart) were used to find out the reliability.

**Results:**

Content validity ratio (CVR), content validity index (CVI), and impact score were 0.98, 0.98, and 3.54 for EBFSS, respectively. This indicates a good content and face validity. Exploratory factor analysis (EFA) was performed on 16 items to examine the construct validity identified emotional, instrumental, and informational factors. These factors explained 59.26% of the cumulative variance. The fit indices (CFI = 0.98، TLI = 0.95، χ^2^/df = 4.20، RMSEA = 0.07 and SRMSEA = 0.05) confirmed the validity of the model in a confirmatory factor analysis (CFA). The internal consistency was examined through Cronbach’s alpha and McDonald’s omega coefficients that were 0.90 and 0.92, respectively. Finally, Repeatability and reproducibility were found 0.97 (95% CI: 0.92 to 0.99) using Intra-class correlation. This shows an appropriate reliability of the instrument.

**Conclusions:**

The research findings indicate that the Persian version of the EBFSS has appropriate psychometric properties for evaluating the social support in Iranian women with EBF. This means healthcare providers can use it for screening social support in EBF. Researchers also can use it as a valid instrument.

## Background

Exclusive breastfeeding (EBF) is a fundamental priority in public health and the most effective preventer of infants early deaths worldwide [1, 2]. It can prevent 800,000 infant and toddlers death in developing countries [3]. EBF is defined as feeding the infant exclusively with the mother’s milk with no solid food and other liquids, even water, except for vitamin and mineral supplements drops or syrups [4].

Mother’s milk is a complex biologic liquid with antibacterial properties, rich in nutrients and antibodies, and an ideal food with maximum qualitative and quantitative nutritional balance [5, 6]. Mother’s milk promotes health in infancy and ensures individual’s survival and health during adolescence, youth, middle age, and even old age [7, 8].

Strong pieces of evidence prove the benefits of breastfeeding for mothers and infants. Such benefits for the infants includes lower respiratory tract, urinary tract, and middle-ear (otitis media) infections [9, 10], fewer digestive system diseases such as diarrhea and atopic dermatitis (eczema) [11]. Building a strong emotional bond between mother and child, less postpartum hemorrhage [12], losing the extra weight more quickly [13], and better family planning [14, 15] are some of its benefits for mothers. It also effectively prevent issues such as breast [16], ovarian [17], and endometrial [18] cancers, metabolic syndrome [19], high blood pressure [20], myocardial infarction [21], diabetes [22], and cardiovascular diseases [23]. According to the results of the National Immunization Survey in the United States, 911 deaths would be prevented if 90% of infants were exclusively breastfed for 6 months [24]. Also, the comparison of 1000 infants fed with breast milk against 1000 infants who were never breastfed, showed that in infants not fed with breast milk, 2033 more visits to the doctor’s office, 609 more prescriptions and 212 more days of hospitalization were required in the first year [25].

EBF level was less than 40% in 2011 and 37% in 2012. By the provisions of the sixty-fifth World Health Organization (WHO) session in 2012, this should reach 50% in 2025 [26]. According to a systematic review, Iranian EBF level is 53% [27]. A 2021 cross-sectional study in East Azerbaijan province, Iran showed that 72% of infants are exclusively breastfeed [28].

WHO strongly recommends starting breastfeeding within the first hour after birth, EBF for 6 months and continuing breastfeeding still age two [29]. A crucial strategy to reach such a goal is identifying factors affecting breastfeeding such as the mother’s age [30], race [31], education level [32], smoking [33], obesity [34], type of delivery, returning to the workplace [35], breastfeeding self-efficacy, and the received social support.

The perceived social support of women has a crucial effect on the length of EBF [36]. Social support as a social network helps individuals overcome life stressful conditions and issues by providing considerable psychological resources [37]. It consists of support behaviors making the individual believe family and friends care for and approve him or her [38, 39]. It can be categorized into instrumental, emotional, informational and appraisal support behaviors [40]. Social support sources are very diverse. Family is the first place to experience social support. Peers, friends, and colleagues are other sources of such support. The lack of familial social support is a big EBF hindrance [41].

A direct relationship and positive correlation have been observed between social support and EBF in developed countries. Some studies have reported an inverse relationship [42]. This can be due to methodological issues or inadequate accuracy of the EBFSS instruments used. Existing instruments’ measurements of social support during breastfeeding focus on any kind of breastfeeding in middle- and high-income countries [43].

Hirari et al., [44] in Pakistan and Zhu et al., [45] in China developed instruments to measure social support during breastfeeding in 2013. Those instruments are not applicable to EBF as they only care about breastfeeding and not about receiving liquids or even other foods. They are not EBF measuring instruments. Focusing of existing instruments on EBF on high-income counties has made determining the relationship between social support and EBF inaccurate. This is implied by the contradictions in the existing studies’ results. Boateng et al. changed this by developing a valid and reliable EBFSS measurement tool in low-income countries in Uganda in 2017. It was 16 items, a 3-point Likert scale measuring emotional, instrumental, and informational social support [46].

Considering that psychometric properties assessment of this tool has not been examined in Iran, and since the most important barriers to breastfeeding in Iran include health care system, physical condition of the mother and finally psychosocial aspects of breastfeeding, including emotional support from husband and family [47], the importance of EBF and the undeniable role of social support in its success and persistence, make it a substantial noteworthy issue. It seems necessary to complete this tool by Iranian women during breastfeeding, with the aim of identifying and screening women with breastfeeding problems, in order to provide interventions. Therefore, in this study we evaluated the psychometric properties of the Persian version of exclusive breastfeeding social support scale (EBFSS) for the first time in Iran.

## Methods

### Research population and setting

This cross-sectional study with descriptive survey method was performed on 348 Iranian clients of health centers associated with the Tabriz University of Medical Sciences between March 2021 and August 2022.

### Validity procedure

Psychometric properties of EBFSS scale were evaluated in six stages: translation, content validity, face validity, construct validity, discriminant validity, and reliability.

### Translation process and testing instrument content validity

After acquiring the permission of Boateng (developer of scale), the scale [46] was translated according to WHO forward-backward protocol [48]. First, two native Persian speakers who were fluent in English and experts in breastfeeding and scales field independently translated the original English scale into Persian. They discussed their translations, resolved conflicts and discrepancies, and provided a final version of translation. In the second step, to ensure its faithfulness and fluency, the final version was created in the Persian language in the previous step, using the backward translation method by two persons who had not seen the original version and were not involved in the translation process of the original version, it was translated into English again. The goal was determining whether the translated items could convey the same meaning as the original items. The back-translated and original English were compared to determine if there’s adequate similarity. Finally, the final version of the translation was given to 10 eligible mothers to check the comprehensibility of questions and concept. The Persian scale was modified according to their comment regarding legibility, grammar, style, and ease of completion [49].

Once the translation was finalized, content validity we examined. It can be defined as the ability of the selected items to reflect the variables of the construct in the measure. It was assessed qualitatively by expert committee method and quantitatively by content validity ratio (CVR) and content validity index (CVI). The scale’s items were put in a content validity assessment form. In qualitative method, 10 experts in reproductive health, midwifery, and nurse education fields were asked to review the scale. For qualitative appraisal, they were supposed to give comment on the content, grammar, phrase length and word count, items ’order, adding new items, and the social and cultural appropriateness of the content. The questionnaire was modified according to these comments [50]. Next, CVR and CVI were used to ensure that question necessity and selecting the best content, respectively. For this purpose, the form consisting of questions in two general sections were given to each expert. The first section assessed CVI according to Waltz and Basel content validity index [51]. A 4-point Likert scale regarding relevance, clarity, and simplicity of each item were designed. For instance the response options regarding relevance were ‘not relevant’, somehow relevant’, ‘relevant’, and ‘totally relevant’. Experts specified their idea about relevance, clarity, and simplicity.

CVI was calculated by the number of specialists who gave 3 and 4 to each items divided by the total number of experts. Items with a CVI more than 0.79 were accepted [52]. To determine CVR using formula $CVR=\frac{Ne-N/2}{N/2}$, we asked 3-point Likert scale questions about the necessity of each item. The response options were ‘necessary’, ‘useful but not necessary’, ‘not necessary’ According to the Lawshe table for number of experts (n = 10), a CVR greater than 0.62 confirmed the necessity of the item [53].

### Face validity assessment

Assuming the target group has the same idea of the rationality of the test as the researcher, the concept means the target group agrees with the phrases used and generality of the instruments. The instruments items must be simple, clear, have an appropriate sequence and font, and an elegant design. This let the target group have no doubt in filling the questionnaire. We studied the face validity with two approaches: qualitative (experts’ and target group’s comments) and quantitative (calculating impact score) [54].

The face validity form contained two sections. First part evaluated it qualitatively by examining levels of difficulty, irrelevance, and ambiguity. The second part (quantitative assessment) calculated the impact score using 5-point Likert scale (‘extremely important’, ‘important’, ‘moderately important’, ‘slightly important’, ‘not at all important’). Next, 10 mothers in their first 4 months after delivery chosen using convenience sampling method were asked to fill the questionnaire. The final impact score for each item was calculated using Impact Score = Frequency (%) × Importance formula [55]. Items with a score of 1.5 or more were declared satisfactory.

### Construct validity assessment through exploratory and confirmatory factor analysis

We determined the construct validity using exploratory factor analysis (EFA) followed by confirmatory factor analysis (CFA). EFA summarizes the data. It puts correlated variables in the same group. CFA tests the existing hypotheses regarding the variables structure [56].

Choosing the necessary sample size for a factor analysis involves many contradictions. A rule of thumb categorizes the sample size for EFA into very poor (50), poor (100), fair (200), good (300), very good (500), and excellent (1000) [57]. The sample size for assessing the construct validity of each item should be 5 to 10 in factor analysis. This enables us to generalize the results to the whole population, for a 16-item EBFSS scale and 10 sample per item, the total sample size became 160. However cluster sampling (due to the intra-cluster correlation effect in cluster sampling) and applying a design effect of 2 increased the sample size to 320. In most situations, the numerical value of the design effect is considered to be about 1.5-2 [58], which we considered 2 in the present study. Assuming a 10% non-response, finally we selected 348 eligible women.

In the present study, in order to carry out the sampling process, in the first stage, using the multi-stage cluster sampling method, half of the 82 health centers of Tabriz city were randomly selected using ‘www.Random.org’ website. Then, a list of women in the first four months after delivery were extracted randomly through the SIB system (integrated health system). The number of women chosen from each center was proportionally calculated concerning the sample size, and they were chosen at random using the same website. The researcher then called participants using their phone number, in case of informed consent and eligibility criteria, the researcher requested them to attend the health centers at the appointed time to complete the questionnaire. The researcher provided those women with comprehensive information after assessing their baseline information and inclusion/exclusion criteria. The inclusion criteria were having a healthy term infant less than four months old, having a case file in health centers, and a vaginal or C-section delivery. Not responding to more than 20% of scale’s questions and a history of traumatic experiences in the last six months, including loss of a first degree relative, would exclude the person from the study.

### Measures

Data collection instrument consisted of two questionnaires. The first questionnaire collected basic personal details of the participants including age, spouse age, number of pregnancies, type of delivery, education level, job of the spouse, income, and history of breastfeeding. The second one was the Persian version of EBFSS scale which content, face, construct, discriminant, and reliability were evaluated in this study. The original scale was designed by Boateng et al. in Uganda in 2017. It contained 16, 3-point Likert scale items that were self-reported measure. The response options were “didn’t help at all” (= 0), “helped less than requested” (= 1), and “helped as requested” (= 2). The respondent has 5 min to fill the questionnaire. Minimum and maximum total scores were 0 and 32. Higher score meant more received social support [46].

Kaiser- Meyer Olkin (KMO) criteria and Bartlett’s sphericity test were used to assess the factor analysis appropriateness of the data in EFA [59]. KMO test is a statistic indicating the proportion of variance among questions due to main factors. A value between 0.8 and 1 means data sample is sufficient for factor analysis. Less than 0.7 value means insufficient sampling and requiring corrective measures [60]. Bartlett’s sphericity test is another fairly standard test to determine data appropriateness in factor analysis. The statistical significance of this tests means the data is suitable for factor analysis [61]. Factor extraction from 16 items of the questionnaire was performed using principal component analysis with varimax rotation (Direct Oblimin) and choosing the number of factors based on Eigenvalue > 1 criterion and scree plot. The minimum factor loading to extract factors in our analysis was 0.3.

CFA uses maximum likelihood methods to estimate the pattern of fit indices and a number of indices to examine the fitness of the pattern. We evaluated the model fitness using the following indices [62, 63]: Root mean score error of approximation (RMSEA < 0.08), standardized root mean square residual (SRMR < 0.10), normed Chi2 (x2/ df) < 5, comparative fit indices including comparative fit index (CFI > 0.90) and Tucker-Lewis Index (TLI) > 0.90.

### Discriminant validity assessment

Discriminant validity is part of construct validity that was studied using known group method [64]. Results of some studies on EBFSS in post-delivery period implies that husbands with higher educations and more income are supposed to provide more social support for their wives. Therefore the independent t-test was used to evaluate the discriminant validity of husband education level and family income in inter-group EBFSS scores. The Cohen’s effect size intervals were determined using $M2-M1/Pooled SD$ equation, according to Cohen’s definition, as 0.2–0.5 (low), 0.5–0.8 (moderate) and > 0.8 (high) [65].

### Reliability assessment

Cronbach’s alpha and Macdonald’s omega coefficients were used to determine reliability of the scale and stability/repeatability was examined using test-retest method [66, 67]. The internal consistency of the instrument was calculated for each subscale and the whole scale using Cronbach’s alpha coefficient. A higher that 0.7 value was considered acceptable. The repeatability of the scale was assessed by having 30 women of randomly sampled of health-centers of Tabriz who were in their postpartum period fill the scale two times, two weeks apart. The correlation between the scores of the two studies was determined using inter-class correlation coefficient (ICC) test and a confidence interval of 95%. Indices higher than 0.6 meant acceptable stability. The results are categorized into poor (0-0.40), fair (0.40–0.59), good (0.60–0.74), and excellent (0.75-1.0) [68].

### Ethical consideration

The current study was approved by the Ethics Committee of Tabriz University of Medical Sciences [IR.TBZMED.REC.1400.772]. The necessary permit was acquired from the designer of the original scale (Boateng) [46] before utilizing the EBFSS questionnaire. All ethical principles including providing comprehensive information about the goals, methods, and reasons of the research to the participants by the researcher, ensuring the participant of the confidentiality of the gathered information, the possibility of exiting the study in any level, and getting an informed written consent were observed.

### Statistical data analysis

Softwares were used to analyze data: SPSS Statistics 14 (IBM Corp, Armonk, NY, USA), STATA 14 (Statcorp, college station, Texas, USA) and R software 4.2 (Psych package). The following descriptive and analytical indices were used to determine the reliability: Mean (SD: standard deviation) for quantitative variables, frequency (percent) for qualitative ones in describing the sociodemographic characteristics, CVR and CVI for content validity, impact score for face validity, independent t-test for discriminant validity, CFA and EFA for construct validity, and ICC, Cronbach’s alpha and Mcdonald’s omega coefficient for reliability.

## Results

A total of 348 women in their post-delivery period chosen by a multi-stage random cluster sampling were present in the study from March 2021 to August 2022. The participants were 16 to 46 years old. The mean (SD) of the participants’ age, number of pregnancies was 30.4 (0.6) and 2.0 (1.0). More than three-quarters (79.3%) had a C-section delivery. Table 1 summarizes other socio-demographic characteristics of the participants.

**Table 1:** Socio-demographic characteristics of participants for factor analysis of EBFSS (n = 348)

Characteristics	Mean	SD
**Age** (Year) **Spouse age** (Year) **Gravidity** **Parity**	30.4 35.5 2.0 1.6	6.0 6.1 1.0 0.6
	**Number**	**Percent**
**Type of delivery**		
NVD CS	72 276	20.7 79.3
**Education level**		
Intermediate or below	77	22.1
Diploma and high school	271	77.9
**Spouse Educational level**		
Intermediate or below	83	23.9
Diploma and high school	265	76.1
**Job**		
Housewife	315	90.5
Employee	33	9.5
**Income**		
Not at all sufficient	53	15.2
Relatively sufficient	229	65.8
Completely sufficient	66	19
**Breastfeeding history**		
Yes	199	57.2
No	149	42.8

Abbreviations: SD, Standard deviation; NVD, normal vaginal delivery; CS, cesarean section

The mean (SD) of EBFSS as a whole was 21.54 (7.27) and for the extracted emotional, instrumental, and informational factors was 3.49 (2.03), 11.73 (3.45), 2.88 (6.32), respectively (Table 2).

**Table 2:** Stability Coefficients and Interclass Correlation Coefficient of the EBFSS (n = 348)

Factors	Mean (SD)	Cronbach’s α coefficient	McDonald’s omega	ICC	CI (95%)
Instrumental	3.49 (2.03)	0.79	0.80	0.93	0.84, 0.97
Emotional	11.73 (3.45)	0.80	0.88	0.93	0.82, 0.97
Informational	6.32 (2.88)	0.82	0.86	0.97	0.92, 0.99
Total score	21.54 (7.27)	0.90	0.92	0.97	0.92, 0.99

Abbreviations: ICC, intra class correlation coefficient; CI, confidence interval

The impact score, CVR, and CVI found to be 0.98, 0.98, and 3.54 in the content and face validity evaluation of the instrument. Table 3 shows the results of content and face validity evaluation.

**Table 3:** The results for the content and face validity of the Iranian version of EBFSS (n = 10)

Item label	CVI	CVR	Impact score
1. Did task	0.96	1.00	4.00
2. Meals	1.00	1.00	4.00
3. Laundry	1.00	1.00	3.50
4. Approved EBF	0.86	1.00	4.00
5. Cared well	0.96	1.00	3.80
6. Feel confident	1.00	1.00	2.70
7. Listened	0.96	0.90	3.10
8. Good mother	0.96	0.90	4.00
9. Concern phy	1.00	1.00	3.10
10. Concern sad	0.96	0.90	4.00
11. Praised EBF	1.00	1.00	4.00
12. Answered Qs	1.00	1.00	2.80
13. Advice EBF	1.00	1.00	4.00
14. Get help	1.00	1.00	3.60
15. Showed EBF	1.00	1.00	2.70
16. Taught care	1.00	1.00	3.40
Total	0.98	0.98	3.54

Abbreviations: CVI, Content Validity Index; CVR: Content Validity Ratio

**Fig. 1:**
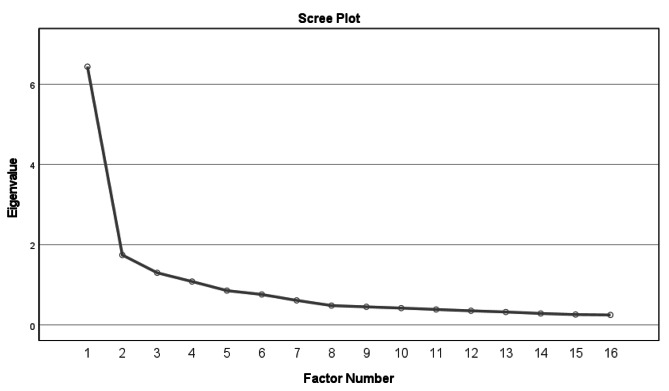
Factor load scree plot of the items for determining the number of extracted factors of the Iranian version of Exclusive breastfeeding social support (EBFSS)

**Fig. 2:**
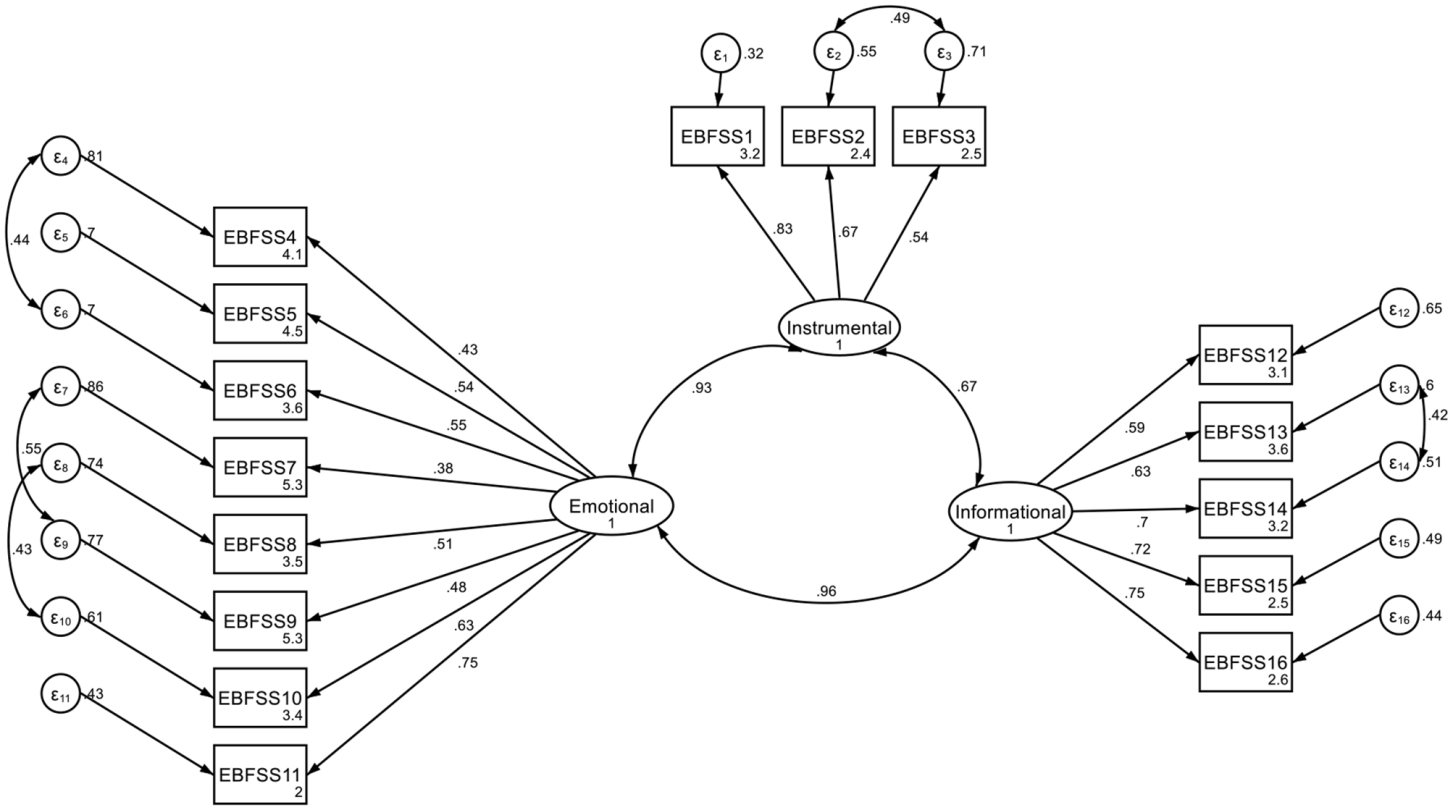
Factor structure model of the EBFSS based on CFA. (All factor loadings are significant at p < 0.001, F1: Instrumental, F2: Emotional, F3: Informational)

In evaluating of the construct validity, the EFA was performed on 16 items. The value of Kaiser-Meyer-Olkin (KMO) was 0.88 with a less than 0.001 significance level. This indicates the sample size was adequate for our study. The Bartlett’s sphericity test was significant which showed an acceptable factor analysis execution considering the correlation matrix in the studied sample (P ≤ 0.001) (Table 4). EFA revealed three factors with eigenvalues greater than one in the Scree plot. These factors explained 59.26% of the variance (Fig. 1). Table 5 shows the extracted variances and the items corresponding to each factor. The first factor was instrumentally received social support. It has 3 questions and a 40.24% share of the total variance. Emotional and informational support, the second and third factors, had 8, and 5 questions and explain 10.90 and 8.12% of the total variance, respectively (Fig. 2).

**Table 4:** KMO and Bartlett’s Test

Measures	Value
KMO Measure of Sampling Adequacy	0.88
Bartlett’s Test of Sphericity Approx	2616.09
Df	120
P-value	0.001>

Abbreviations: KMO, Kaiser- Meyer Olkin; df, degree of freedom

**Table 5:** Facture structure of the EBFSS scale based on EFA (n = 348)

Scale item	Item label		Factors	
		1	2	3
**Factor 1: Instrumental**				
1. Did tasks I would normally do so that I could exclusively breastfeed	Did task	0.617		
2. Prepared meals	Meals	0.889		
3. Did laundry	Laundry	0.751		
**Factor 2: Emotional**				
4. Approved of me exclusively breastfeeding my baby	Approved EBF		0.452	
5. Told me I was doing well caring for my baby	Cared well		0.699	
6. Made me feel confident even when I made mistakes	Feel confident		0.495	
7. Listened to me talk about the new baby	Listened		0.566	
8. Believed that I am a good mother	Good mother		0.640	
9. Showed concern about my own physical condition and health	Concern phy		0.676	
10. Showed concern when I felt sad or depressed	Concern sad		0.723	
11. Praised me for my efforts to exclusively breastfeed	Praised EBF		0.503	
**Factor 3: Informational**				
12. Answered Qs Answered my questions about breastfeeding	Answered Qs			0.533
13. Advice EBF Gave me advice and suggestions about how to exclusively breastfeed	Advice EBF			0.701
14. Get help Told me where I could get help if I had questions about breastfeeding or caring for my baby	Get help			0.795
15. Showed EBF Showed me how to breastfeed	Showed EBF			0.714
16. Taught care Taught me how to take care of myself	Taught care			0.707
**% of variance observed**		40.24	10.90	8.12
**Total score**	59.26			

The three factors found in EFA were also examined in CFA. Our result shows this model achieves an appropriate fitting level. This can be used to confirm the factor structure.

The index was 4.203 (χ2 = 403.582, df = 96, P-value < 0.001), the Tucker-Lewis fitting indices (TLI) and CFT were more than 0.9, and root mean squared error of approximation (RMSEA) and standardize mean squared residual (SRMR) were 0.670 and 0.530, all of them confirming the validity of the model (Table 6).

**Table 6:** The model fit indicators of the EBFSS (n = 348)

Goodness of fit indices	CFA	Acceptable value
**χ2** **df**	403.582 96	
$$\raisebox{1ex}{${\varvec{x}}^{2}$}\!\left/ \!\raisebox{-1ex}{$\varvec{d}\varvec{f}$}\right.$$	4.203	< 5
**P-value**	< 0.001	0.05>
**CFI**	0.979	> 0.90
**TLI**	0.949	> 0.90
**SRMR**	0.053	< 0.10
**RMSEA (90% CI)**	0.076 (0.067, 0.086)	< 0.08

Abbreviations: χ2, chi-square; df, degrees of freedom; χ2/df, normed chi-square; CFI, Comparative Fit Index; TLI, Tucker–Lewis index; SRMR, Standardized root mean squared residual; RMSEA, root mean square error of approximation

We used known group method to examine the discriminant validity as part of the construct validity. In men with higher education levels the overall score of EBFSS and the score for instrumental subdomain was significantly higher than men with lower education level with low effect size. The overall EBFSS score and instrumental and informational subdomain scores were also significantly different between women with insufficient and sufficient incomes (Table 7).

**Table 7:** EBFSS overall and sub scales scores by different groups (n = 348)

Variables	Instrumental	Emotional	Informational	Total scale
		Mean (SD)		
**Spouse Education**				
Low school (n = 83)	2.9 (1.9)	11.1 (3.8)	5.6 (2.9)	20.0 (7.6)
High school (n = 265)	3.7 (2.0)	11.9 (3.3)	6.4 (2.9)	22.0 (7.1)
**P-value**	0.001	0.112	0.216	0.034
**Cohen’s effect size**	0.41	0.22	0.27	0.27
**Income**				
Sufficient (n = 295)	3.6 (2.0)	11.8 (3.5)	6.5 (2.9)	21.9 (7.3)
Insufficient (n = 53)	2.8 (2.0)	11.1 (3.3)	5.5 (2.9)	19.4 (7.0)
**P-value**	0.015	0.173	0.024	0.022
**Cohen’s effect size**	0.40	0.17	0.34	0.56

Finally the Cronbach’s alpha and Macdonald’s omega coefficient were 0.90 and 0.92 in determining reliability. This indicated appropriate internal consistency of the questionnaire. Assessing stability and repeatability of the instrument using test-retest method gives us an ICC (Confidence interval 95%) level of 0.97 (0.92 to 0.99) (Table 2).

## Discussion

EBF is a key concept in achieving the third goal of sustainable development, i.e., eradicating preventable neonate’s deaths by 2030 and a fundamental priority of public health worldwide [69]. Nevertheless, despite the repeated emphasis of WHO on promoting EBF only 43% of less than six months neonates are exclusively breastfeed [70]. The significance and clinical consequences of the EBF in developed and developing countries makes identifying the existing obstacles in this field significant [71].

Considering the loss of physical and psychological strength of mothers in postpartum period, receiving social support from family, friends, and health-care providers is very important in the success of their EBF [72]. A study in north Ethiopia indicated that socially supported mothers were four times more successful in EBF [73]. Another study showed that women who had prematurely terminated their breastfeeding were about 22 times less socially supported than breastfeeding mothers [74].

This makes screening mothers’ received social support during EBF useful, which requires measurement with valid and reliable scales. Our study aimed to find the psychometrics of EBFSS scale in Iranian women. The results indicate that the Persian version of the questionnaire can be a valid and reliable instrument to evaluate the received social support during EBF in Iranian women due to its psychometric properties.

This is the first scale specifically developed and applicable to low-resource countries [46]. The EFA extracted informational, instrumental, and emotional factors for the 16-item scale that explained about 60% of the variance. That value was 66% in the original scale. The value of KMO test and the significance of Bartlett’s test also confirmed the adequacy of the model. Factors extracted during EFA were consistent with the three factors reported by Hughe (1984) social support during breastfeeding period. EBFSS (16 questions) advantage is needing less time to fill than Hughe scale (30 questions) [75].

The total mean (SD) score of EBFSS in our research was 21.54 (7.27) with a Cronbach’s alpha value of 0.90. The reported values for these parameters in Boateng et al. study were 19.1 (4.2) and 0.9. The Cronbach’s alpha in that case was 0.86 and between 0.78 and 0.85 for three factors. Our scale extracted factors confirm the results of some of the existing studies in this area. A systematic review study in 2022 indicated that social support as an influential factor in persistence and success of the EBF has four dimensions: emotional support, appraisal support, informational support and material and service support and it should be promoted individually or collectively from pregnancy to postpartum period to promote EBF [76]. Fadjriah et al. [77] in a cross-sectional study identified emotional, instrumental, informational, and appraisal dimensions which were consistent with the three extracted factors of our study. It demonstrated that all four dimensions have a significant relationship with EBF and not receiving social support is a substantial obstacle that leads to EBF failure [77].

Informational social support, a crucial dimension of social support, consists of the information that the family or healthcare providers present to mother to support her success in EBF. Bich et al. educated fathers on EBF to promote supporting the wives. Their result after one year of intervention showed that the intervention group mothers (49.2%) were more probably begin breastfeeding than control group mothers (35.8%). A mother’s knowledge of and education level on EBF advantages might persuade the mother to EBF her child [78].

Instrumental social support, another significant factor in social support that is consistent with our study, is about the correct fundamental way of breastfeeding and directly providing facilities to support the success of the EBF [79]. Mothers not receiving assistance from their family experience breastfeeding difficulty. Emotional Social support, the third dimension, is expressing sympathy that enhances the trust of the mother by the family to reduce her stress and bring comfort and peace of mind. A study demonstrated that depressed mothers were four times more likely refrain from EBF than non-depressed mothers. Also, shorter EBF period was comorbid with postpartum depression [80].

The low EBF rate, the relationship between EBF and preventable neonate’s deaths, and the proven relationship between EBF and the social support received from the healthcare providers and family, and the negative impact of COVID-19 pandemic highlight the significance of a special instrument to measure received social support during breastfeeding.

### Strengths and limitations

Examining the psychometric properties of EBFSS scale for the first time in Iran, random selection of participants, using the forward-backward method according to the WHO protocol for translation process, including all women with vaginal and C-section delivery history, and being practical in countries with any level of resources are among the strengths of our study. The present study also had some limitations that need to be mentioned. First, the potential bias due to a tendency to give desired responses with self-reported measures. Second, performing EFA and CFA on a same data set. Third, the lack of calculation of criterion validity due to the absence of a gold standard for measuring EBFSS. Finally, due to the cross-sectional nature of this study, it is not possible to determine the causal relationships between social support and other indicators of exclusive breastfeeding.

## Conclusions

The research finding indicates that the Persian version of the EBFSS has appropriate psychometric properties for evaluating the social support in EBF Iranian women. This means healthcare providers can use it for screening social support in EBF. Researchers also can use it as a valid instrument. Therefore, health policy maker should pay special attention to EBF. They should do their best to plan special programs that educate healthcare providers and families on providing appropriate social support to promote EBF performance in mothers.

## Data Availability

The datasets generated and/or analysed during the current study are not publicly available due to limitations of ethical approval involving the patient data and anonymity but are available from the corresponding author on reasonable request.

## Abbreviations

EBFSS: Exclusive breastfeeding social support
EBF: Exclusive breastfeeding
EFA: Exploratory factor analysis
CFA: Confirmatory factor analysis
ICC: Intra-class Correlation Coefficient
SD: standard deviation
CVI: Content validity index
CVR: Content validity ratio
df: degree of freedom
KMO: Kaiser-Meyer-Olkin
RMSEA: Root mean squared error of approximation
CFI: Comparative fit index
TLI: Tucker–Lewis index
